# Upregulation of GTPBP4 Promotes the Proliferation of Liver Cancer Cells

**DOI:** 10.1155/2021/1049104

**Published:** 2021-10-19

**Authors:** Jia Chen, Jie Zhang, Zhiwei Zhang

**Affiliations:** ^1^Cancer Research Institute of Hengyang Medical College, University of South China, Hengyang, China; ^2^Physical Examination Center, Shanghai Tenth People's Hospital, Tongji University, Shanghai, China; ^3^Department of Laboratory Medicine, Shanghai Tenth People's Hospital, Tongji University, Shanghai, China

## Abstract

**Results:**

The GTPBP4 has upregulated expression in liver cancer patients (*P* < 0.01), but there was no difference in its expression in patients with different clinicopathological stages. The expression of GTPBP4 increased with the increase of cancer metastasis in lymph nodes (*P* < 0.01). Liver cancer patients with upregulated expression of GTPBP4 showed a shorter overall survival rate (*P*=0.02). GTPBP4 is closely related to genes such as NIFK, WDR12, and RPF2, and these genes are involved in life processes such as GTP binding and rRNA processing. The upregulated expression of GTPBP4 promotes the proliferation of liver cancer cells and promotes the growth of tumors in mice, while the downregulated expression of GTPBP4 inhibits the proliferation of liver cancer cells and inhibits the growth of tumors in mice.

**Conclusion:**

The expression of GTPBP4 is upregulated in liver cancer patients and affects the overall survival rate of patients. The upregulated expression of GTPBP4 promotes the proliferation of liver cancer cells and the growth of tumors.

## 1. Introduction

Liver cancer is a public health issue worldwide and ranks second in cancer-related mortality rate worldwide [1]. In China, liver cancer is the fourth most prevalent type of cancer, but its mortality rate ranks second among all cancer types [2, 3]. Hepatocellular carcinoma is a popular subtype of liver cancer, accounting for approximately 85–90% of primary hepatic carcinoma. In the Chinese population, hepatitis B virus infection is the main factor leading to the occurrence of hepatocellular carcinoma, but in Western countries, the occurrence of hepatocellular carcinoma is mostly due to hepatitis C virus infection [4]. The five-year survival rate of liver cancer patients is only 18%. After resection, the five-year survival rate of patients can exceed 50% [5, 6]. At present, surgical resection is still the main treatment means for primary hepatic carcinoma. However, the 5-year recurrence rate after surgery is still as high as 70%, and the recurrence of cancer often occurs in the second year after the resection is completed [7]. Therefore, the research on the occurrence and progression mechanism of liver cancer is still urgent.

In human cells, guanosine monophosphate (GMP), guanosine diphosphate (GDP), and guanosine triphosphate (GTP) constitute the basic building blocks of signal transduction and thus become the central link of most cellular processes. GTP binding proteins include membrane protein, tubulin, motility protein, eukaryotic transcription initiation/elongation factor, and small guanosine triphosphatase of the Ras superfamily [8]. More and more evidence indicate that small GTPases and their regulatory factors may promise to become potential therapeutic targets for cancer [9]. According to the sequence homology and physiological function of small GTPases, they can be divided into five categories [10]: Arf subfamily, Ras subfamily, Ras homology (Rho) subfamily, Rab subfamily, and Ras-related nuclear protein (Ran) subfamily [11, 12]. The dysfunction of small molecule GTPases, such as members of the Ras and Arf subfamilies, is related to the occurrence and development of tumors. Therefore, the development of inhibitors for dysfunctional small GTPases may represent a potential cancer treatment strategy. In recent years, the abnormal expression of small GTPases and their abnormal activities have been shown to be closely related to a variety of cancers. For example, in colon cancer, TIF-90 combined with GTP promotes the synthesis of rRNA and thus affects the development of cancer [13], and CRMP5-associated GTPase (CRAG) can upregulate the expression of c-Jun gene, significantly enhancing the proliferation and cloning ability of cancer cells [14]; in liver cancer, the upregulated expressions of RhoA and Rac1, members of the Rho GTPase family of GTP binding proteins, are closely related to the occurrence and development of cancer; in prostate cancer, SEC can induce the activation of ANXA7GTPase, thereby inhibiting cancer metastasis [15]; in breast cancer, the upregulated expression of Ran-GTPase components can regulate nuclear export but cannot affect mitotic spindle assembly, which can indicate chromosomal instability and poor prognosis [16], and ANX7-GTPase can significantly enhance the metastatic ability of cancer cells [16]. Gene mutations can cause abnormal protein activity. KRas has a higher mutation frequency in pancreatic cancer and colorectal cancer [17]. Oncogenic mutations in the Ras gene can impair GAP-mediated GTP hydrolysis and abnormal cell signaling [17].

The main research object of this article is GTP binding protein 4 (GTPBP4), which is a GTPase. GTPase acts as a molecular switch and can switch between two states: active when binding with GTP and inactive when binding with GDP. In this case, “active” usually means that the molecule acts as a signal to trigger other events in the cell. When the extracellular ligand binds with the receptor linked to the *G* protein, the receptor changes its conformation, makes trimeric *G* protein expel its GDP, and uses GTP to replace its related trimeric *G* protein. When *G* protein hydrolyzes the GTP bound with itself and converts it back to GDP, the switch is turned off. But before that, the active protein has the opportunity to diffuse out of the receptor and transmit its information to downstream target cells for a long time. GTPase interacts with a variety of effectors and regulates a variety of different biological pathways, including pathways that regulate cytoskeleton, cell motility, cytokinesis, proliferation, apoptosis, transcription, and nuclear signal transduction. GTPBP4 plays an important role in the biosynthesis of 60s ribosomes [18]. According to reports, in colorectal cancer, GTPBP4 can weaken the activity of RhoA to destroy the skeleton of actin and promote the metastasis of cancer cells [19]. But GTPBP4 is rarely studied in liver cancer. Therefore, this study takes GTPBP4 as the main research object, which can make some explorations for its research in liver cancer.

This study explored the expression of GTPBP4 gene in liver cancer patients and its prompting effect on the prognosis of liver cancer patients in GEPIA2, Kaplan–Meier plotter, and other databases. The genes that interact with GTPBP4 was explored on the STRING website, and functional enrichment analysis was performed on them. Subsequently, the expression of the mRNA level and protein level of the GTPBP4 gene in liver cancer cells was verified by fluorescence quantitative PCR and Western blotting experiments. Through the CCK-8 experiment, the effect of GTPBP4 gene expression on the growth of liver cancer cells was explored. Through nude mouse tumorigenicity assay, the effect of GTPBP4 expression on tumor growth in vivo was explored.

## 2. Materials and Methods

### 2.1. GEPIA2 Database Analysis

GEPIA2 (Gene Expression Profiling Interactive Analysis 2, http://gepia2.cancer-pku.cn/) is a very valuable and highly cited online tool based on TCGA and GTEx databases. It can satisfy various analyses related to the gene expression values of tumor and normal samples, such as survival analysis and differential expression analysis [20]. In this study, it was used to compare the expression of GTPBP4 in liver cancer tissues and adjacent tissues, as well as its expression in different clinicopathological stages. The “survival analysis” function of the GEPIA2 database was used to compare and analyze the overall survival of people with high GTPBP4 expression and low GTPBP4 expression.

### 2.2. Kaplan–Meier Plotter Database Analysis

Kaplan–Meier plotter (http://kmplot.com/private/) database is based on GEO, EGA, and TCGA databases, including 54,000 genes such as mRNA, miRNA, and protein, and covering 21 cancer types such as liver cancer, lung cancer, and breast cancer. It aims to discover and verify some markers with the prognostic value. In this study, it was used to explore the prognosis differences of patients with high GTPBP4 expression and low GTPBP4 expression.

### 2.3. Oncomine Database Analysis

Oncomine (http://www.oncomine.org/resource/main.html) is an online data mining platform based on cancer microarrays, which aims to promote the exploration and discovery of whole-genome expression analysis [21]. One or more selected genes can be queried and visualized in single or multiple cancer types. In this study, this database was used to explore the expression of GTPBP4 in cancer tissues and adjacent tissues of different cancers.

### 2.4. UALCAN Database Analysis

The UALCAN (http://ualcan.path.uab.edu/index.html) database is constructed based on TCGA transcriptome data and patient clinical information. Not only can it help to compare the gene expression differences of patients' primary lesion and metastases but also the gene expression differences in different tumor staging, different races, and samples of clinical cases with different characteristics (such as smoking history). At the same time, it also provides quick links to other related websites, such as GeneCards and TargetScan [22]. This study is used to explore the expression differences of GTPBP4 gene in liver cancer patients with different degrees of lymph node metastasis.

### 2.5. STRING Database Analysis

The STRING database (https://string-db.org/) aims to collect, evaluate, and integrate all public protein interaction information, make up for the deficiencies of these data through computational predictions, and finally construct a comprehensive and objective protein interaction network. The interaction relationships between proteins include not only direct (physical) relationships but also indirect (functional) interaction relationships [23]. This study used STRING to explore genes that have direct and indirect interactions with GTPBP4 and conduct gene function and pathway enrichment analysis of the abovementioned GTPBP4 and its interaction proteins.

### 2.6. Cell Lines and Cell Culture

HepG2 cell line was purchased from American Type Culture Collections (ATCC, USA). Cultured the cells in CM1-1 culture solution containing 90% DMEM-H+10% FBS at 37°C, 5% CO_2_. DMEM-H, DMEM high glucose culture solution, contains glutamine and sodium pyruvate.

### 2.7. Transfection and Overexpression Experiments

Obtained the lentivirus-GTPBP4 (shGTPBP4) for overexpression and the small RNA (si-GTPBP4) that interfere with the expression of GTPBP4 from GeneChem (Shanghai, China). Seeded the cells in a 6-well plate at a concentration of 2 × 10^5^ per well. Each well contained 2 ml RPMI 1640 medium. Two hours before transfection, washed the cells twice with phosphate buffered saline (PBS) and placed them in the Opti-MEM I reduced serum medium (Invitrogen, USA). Added 1 mg of transfectant and 2 ml of Lipofectamine 2000 (Invitrogen, USA) to 250 ml of Opti-MEM, respectively. 5 minutes later, mixed the diluted transfectant and Lipofectamine 2000 and cultured for 30 minutes. Then, added 500 ml transfectant-lipid complex and 500 ml Opti-MEM to the cells in each well. After incubating for 12 hours, added 2 ml of RPMI 1640 with 10% serum to each well. All operations in the control group were the same as those in the experimental group, just replacing the transfectant with the same amount of PBS solution.

### 2.8. Western Blotting Analysis

Harvested the cells when the cell fusion rate reached 70–80%, aspirated the cells of different transfection groups, and placed them in RIPA buffer (150 mM NaCl, 1% NP-40, 0.5% DOC, 0.1% SDS, 50 mM Tris -HCl pH 8.0). After resuspension, added protease inhibitor cocktail (Sigma, USA). Used the Bradford reagent (BioRad, NY) to quantify the protein. Added 25 *μ*g of protein to 10% SDS-PAGE (BioRad) gel wells and performed electrophoresis. After electrophoresis, transferred the protein to a polyvinylidene fluoride membrane. Cultured the nonspecific binding sites in 5% skimmed milk powder PBS for 1 hour to block and then washed with 0.1% Tween-20. Diluted GTPBP4 by 1 : 3000 (EPR3500, Abcam, USA) and then placed it on the membrane for incubation overnight. After washing, incubated the membrane with 1 : 5000 diluted anti-rabbit secondary antibody (conjugated with horseradish peroxidase). Finally, added Western blotting luminescence reagent and imaged in Kodak X-Omat blue film.

### 2.9. Fluorescence Quantitative PCR

Used TRIzol (Invitrogen, USA) to extract RNA from HepG2 cells of different transfection groups and reverse transcribed into cDNA. Used a TaqMan probe (Applied Biosystems, USA) to conduct quantitative real-time polymerase chain reaction. GAPDH, as an internal reference, was used to detect the mRNA expression of GTPBP4. According to the instructions of SYBR Green Master Mix (ThermoFisher Scientific, USA), the experiment was performed on the ABI Prism 7500 detection system (Applied Biosystems, Inc.). GTPBP4 upstream primer: 5′-AGTTGCTCTCGAACTCCACG-3′, downstream primer: 5′-TGTCTATCCGCCTCCCCTTT-3'. GAPDH upstream primer: 5′-GCCTCAAGACCTTGGGCTGGGACTG-3′, downstream primer: 5′-CTAAGTCCCTCCTACAAAA-3'.

### 2.10. CCK-8 Experiment

Collected the stable cells whose GTPBP4 were upregulated or downregulated after transfection. Subsequently, transplanted HepG2 liver cancer cells into a 96-well plate and cultured in the DMEM medium with 2% FBS. Used the cell counting kit-8 (CCK-8) (Dojido, Japan) to detect the OD value of cells on the 1^st^, 2^nd^, 3^rd^, 4^th^, and 5^th^ day according to the instructions. Added 100 *μ*l of free medium containing 10% CCK-8 solution to each well and then cultured for 1–4 hours in humid environment at 37°C and 5% CO_2_. Finally, measured the solution at 450 nm with the spectrophotometric method.

### 2.11. Nude Mouse Tumorigenicity Assay

A total of 18 BALB/C male mice were included in this study, and they were randomly divided into three groups with 6 mice in each group. The three groups are control, GTPBP4 (GTPBP4 overexpression), and shGTPBP4 (GTPBP4 downexpression) groups. After mixing 1 × 10^6^ transfected cells of different groups with PBS, injected the solution into the mice through subcutaneous injection in the back and neck areas. The GTPBP4 group was injected with GTPBP4 overexpressed cells, and the shGTPBP4 group was injected with GTPBP4 downexpressed cells. The control group was injected with equal volume of PBS solution. All mouse was raised in same environment. Measured the volume of mouse tumors every 4 days. At 24^th^ day, collected and weighed the tumors of different transfection groups.

### 2.12. Statistics and Analysis

All the experiments were carried out for at least three times, and all the data were expressed as mean ± standard deviation. All the differences between the two groups were analyzed by the *t*-test.

## 3. Results

### 3.1. GTPBP4 Expression and Prognostic Analysis in Patients with Liver Cancer

**Figure 1:**
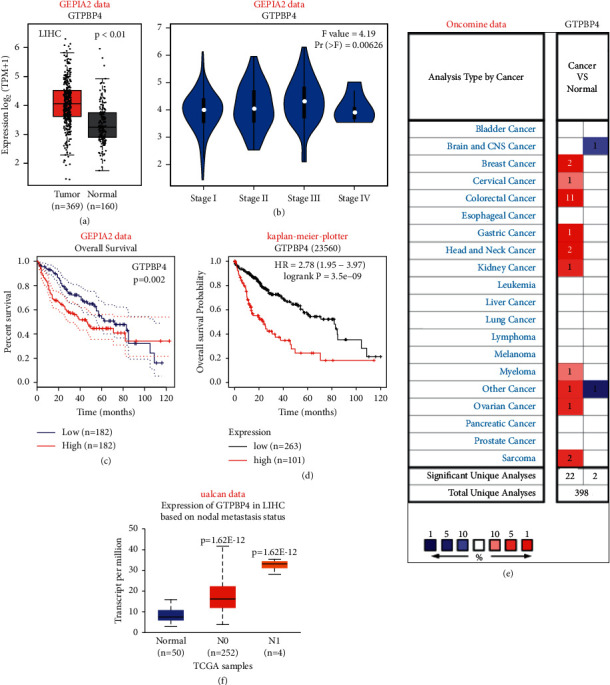
(a-b) The GEPIA database showing the expression of GTPBP4 in liver cancer and normal liver tissues (a) and the expression in different pathological stages (b). (c-d) GEPIA and Kaplan–Meier plotter databases showing the relationship between the GTPBP4 expression level and the prognosis of patients with liver cancer. (e) Oncomine database showing the expression of GTPBP4 in different tumors. (f) The UALCAN database showing that the expression level of GTPBP4 is related to whether the patient has lymph node metastasis.

In order to observe the expression of GTPBP4 in liver cancer patients and the overall survival of liver cancer patients with different expression levels of GTPBP4, we searched GTPBP4 in various online databases. The results showed that GTPBP4 had a high expression in liver cancer patients (Figure 1(a), *P* < 0.01). There was no difference in the expression of GTPBP4 in liver cancer patients with different clinicopathological stages (Figure 1(b)). However, in patients grouped only by the distance to lymph node metastasis, GTPBP4 increased with the degree of lymph node metastasis, and the expression level increased (*P* < 0.05, Figure 1(f)). The overall survival rate results showed that liver cancer patients with downregulated expression of GTPBP4 had a better overall survival rate (Figures 1(c) and 1(d)). Subsequently, we observed the expression of GTPBP4 in different cancer types. The results showed that GTPBP4 had an upregulated expression in most cancer types (Figure 1(e)).

### 3.2. GTPBP4 Function and Pathway Analysis

**Figure 2:**
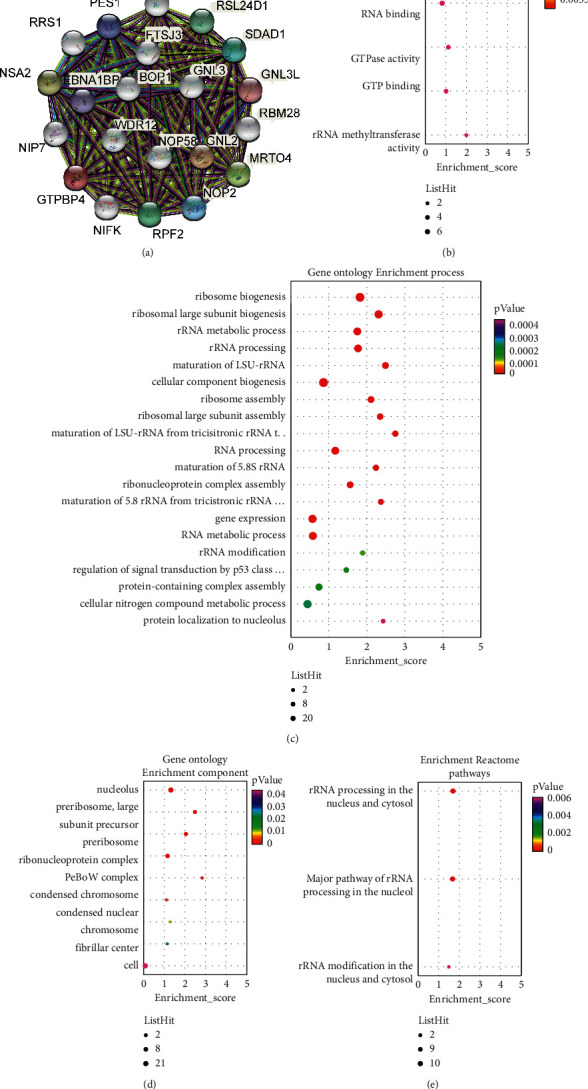
(a) STRING database analyzing GTPBP4 protein interaction network diagram, (b–d) Gene ontology (GO) function analyzing the molecular functions, cell components, and participant biological processes of GTPBP4 interacting proteins with annotation. (e) Reactome pathway analyzing the related signaling pathways involved in GTPBP4 interacting proteins.

In order to further explore the proteins that can interact with GTPBP4, as well as their functions and pathways in life body, we constructed a protein interaction network of GTPBP4 and conducted an enrichment analysis of their gene functions and signal pathways. The protein interaction network results showed that GTPBP4 had an interaction relationship with genes such as NIFK, NIP7, RPF2, and WDR12 (Figure 2(a)). The results of gene function showed that GTPBP4 and its interacting proteins were related to the biosynthesis of ribosome, participating in processes such as 5s rRNA binding, ribosome biogenesis, and rRNA modification (Figures 2(b)–2(d)). Participating in RNA binding affected gene expression (Figure 2(b)). It was also involved in signal transfer mediated by the p53 family (Figure 2(c)). The results of pathway analysis showed that GTPBP4 and its interacting proteins were mainly involved in some pathways related to rRNA, such as rRNA processing in the nucleus and cytosol (Figure 2(e)).

### 3.3. In Vitro and In Vivo Experiments to Explore the Effect of GTPBP4 on Liver Cancer Cells

**Figure 3:**
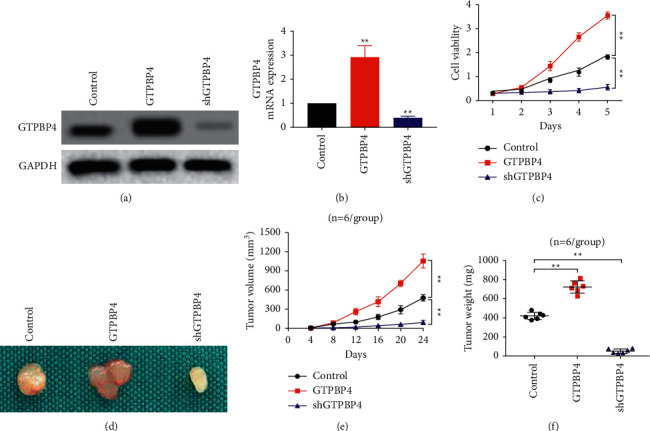
(a-b) WB and RT-qPCR methods detecting the overexpression and knockdown efficiency of GTPBP4. (c) Changes of cell proliferation after overexpression and knockdown of GTPBP4 in HepG2 cells. (d) Subcutaneous xenograft tumor size of overexpression and GTPBP4 knockdown on nude mice. (e-f) Tumor growth curve and tumor weight of subcutaneous xenograft tumor.

We used cell experiments to explore the effects of overexpression and underexpression of GTPBP4 on liver cancer cells. The results of Figure 3(a) and Figure 3(b), respectively, show the protein and mRNA expression of GTPBP4 in the overexpression group and the inhibited expression group, verifying the success of the transfection experiment. The results of cell proliferation experiments showed that overexpression of GTPBP4 could promote the proliferation of liver cancer cells and that inhibiting the expression of GTPBP4 could also inhibit the proliferation of liver cancer cells (Figure 3(c)). In order to further explore the effect of GTPBP4 on liver cancer cells in vivo, we conducted the nude mouse tumorigenicity assay. The results showed that the tumor volume in the GTPBP4 overexpression group was significantly increased, while the tumor volume in the GTPBP4 inhibited expression group was significantly reduced (Figures 3(d) and 3(e)). At the same time, the tumor weight in the GTPBP4 overexpression group also increased significantly and decreased in the GTPBP4 inhibited expression group (Figure 3(f)).

## 4. Discussion

In recent years, research studies on GTP binding proteins have been increasing. As one of the GTP-binding proteins, the small GTPase plays an important role in signal transduction and transfer. Therefore, the exploration of the mechanism of small GTPases will help to study their role in the occurrence and development of diseases, thereby, providing more options for disease treatment [23]. This study found that GTPBP4 had an upregulated expression in liver cancer patients by mining public databases (*P* < 0.01), and its expression level increased with the increase of cancer metastasis in lymph nodes (*P* < 0.01). It was reported that in lung adenocarcinoma, colorectal cancer, and breast cancer, GTPBP4 showed a trend of upregulated expression [19, 24, 25]. In addition, we also found that liver cancer patients with upregulated expression of GTPBP4 showed a shorter overall survival time (*P*=0.02), which is consistent with the results of previous studies [26, 27]. The expression of GTPBP4 was positively correlated with the invasion of B cells and macrophages, which may be one of the reasons why the expression of GTPBP4 can affect the overall survival time of liver cancer patients. Besides, the researchers also found that GTPBP4 gene mutations could promote the proliferation of melanoma cells [28], and the expression of GTPBP4 was also closely related to the clinical stage of bladder cancer, cancer metastasis, and the overall survival time of patients after surgery.

According to the results of the STRING database, GTPBP4 has a functional or structural interaction with genes such as NIFK, WDR12, and RPF2. NIFK activates TCF4/*β*-catenin signaling and Ki-67-dependent cell proliferation regulation through RUNX1-dependent CK1*α* repression and participates in the progression of lung cancer [29]. In addition, the RNA recognition motif of NIFK is necessary for rRNA maturation during the cell cycle process [30]. RPF2 is necessary for the maturation of the 60s ribosomal subunit [31]. The long chain noncoding RNA LINC00963 promotes the metastasis of prostate cancer through regulating the expression of NOP2 by miR-542-3p [32]. MRT4 homolog, ribosome maturation factor (MRTO4) is a transacting factor involved in the maturation of ribosomes and has the ability to shuttle between the nucleus and cytoplasm [33]. In the process of tumorigenesis, GNL3L-LDOC1 regulates cell proliferation by regulating the NF-kappaB pathway [34], and the absence of GNL3L can also impair the production of ribosomes [35]. MiRNA-378 can inhibit the expression of SDAD1 to inhibit the proliferation, migration, and invasion of colorectal cancer cells [36]. The protein encoded by RSL24D1 is located in the nucleolus and plays a role in the biogenesis of the 60s ribosomal subunit. CD44 regulates the expression of PES1 by regulating miR-105-5p and promotes the proliferation of liver cancer cells [37, 38]. Interfering with the expression of RRS1 can promote the apoptosis of liver cancer cells and inhibit the proliferation of liver cancer cells [39]. NSA2 (also called TINP1) can inhibit the expression of p53 and p21, thereby promoting cell proliferation [40]. Besides, NSA2 may also be involved in the regulation of ribosomal biosynthesis [41]. In liposarcoma, NIP7, RPL10L, and MCM2 are significantly associated with long-term no recurrence survival rate [42]. There is a close functional interaction between NIP7 and FTSJ3 during pre-rRNA processing, which indicates that FTSJ3 is involved in ribosome synthesis in human cells [43]. BOP1 promotes the transformation of epithelial cells to mesenchymal cells and exerts a carcinogenic effect in hepatocellular carcinoma [44]. GNL3 promotes the transformation of colon cancer epithelial cells to mesenchymal cells by activating the Wnt/*β*-catenin signaling pathway. GNL3 has upregulated expression in colon cancer and plays an important role in tumor growth, invasion, and metastasis [45]. Interfering with the expression of WDR12 can inhibit the cell cycle, thereby inhibiting glioma [46]. In addition, the interdependence of Pes1, Bop1, and WDR12 controls the nucleolar localization and assembly of the PeBoW complex required for 60s ribosomal subunit maturation [47]. The long chain noncoding RNA FAM83A-AS1 enhances the stability of FAM83A mRNA by binding to NOP58 and promotes the progression of hepatocellular carcinoma [48]. NGP-1 promotes cell cycle progression by activating the p53/*p*21 (Cip-1/Waf1) pathway [49]. It can be seen that most of the proteins that interact with CTPBP4 are closely related to the proliferation or apoptosis of cancer cells and are also involved in the biosynthesis of ribosomes. This is consistent with the results of gene function and pathway enrichment analysis. Although the causal relationship between genetic mutations that affect ribosome biogenesis and increased cancer risk has been established in the past decade, mechanistic data indicate that ribosome biogenesis dysregulation plays a wider role in the development and progression of most spontaneous cancers [50]. It suggests that GTPBP4 may affect cancer cells by affecting the biosynthesis of ribosomes.

In order to verify the effect of GTPBP4 gene on the proliferation of liver cancer cells in vivo and in vitro, this study conducted CCK-8 experiments and nude mouse tumorigenicity assay. Experimental results showed that the upregulated expression of GTPBP4 promoted the proliferation of liver cancer cells and promoted the growth of tumors in mice and that the downregulated expression of GTPBP4 inhibited the proliferation of liver cancer cells and inhibited the growth of tumors in mice. This indicates that GTPBP4 is not only related to immune cell invasion, affecting the prognosis of liver cancer patients, but also directly affects the proliferation of liver cancer cells, promoting the development of liver cancer. However, this study still has some shortcomings, that is, it failed to test the signal pathway of GTPBP4 gene affecting the proliferation of liver cancer cells.

In summary, this study found that GTPBP4 is highly expressed in liver cancer patients and is significantly related to the degree of lymph node metastasis through our exploration of public databases. The upregulated expression of GTPBP4 will reduce the overall survival rate of liver cancer patients. In addition, the results of the protein interaction network and functional enrichment analysis show that GTPBP4 is involved in signal transduction processes such as ribosome biosynthesis. The upregulated expression of CTPBP4 can also promote the proliferation of liver cancer cells in vivo and in vitro.

## Data Availability

The data used to support the findings of this study are available from the corresponding author upon request.
